# Real-world status of continuous positive airway pressure (CPAP) persistence in patients with sleep apnea syndrome (SAS): a retrospective longitudinal study of administrative claims data in Japan

**DOI:** 10.1007/s11325-025-03417-w

**Published:** 2025-07-17

**Authors:** Hiroyuki Takahashi, Shiori Yoshida, Akihiro Nakajima, Ruriko Koto, Hideaki Nakayama

**Affiliations:** 1 Medical Science Department, Teijin Pharma Limited, Tokyo, Japan; 2 Clinical Development Control Department, Teijin Pharma Limited, Tokyo, Japan; 3 Department of Somnology, Tokyo Medical University Hospital, Tokyo, Japan

**Keywords:** Continuous positive airway pressure, Persistence, Real-world data, Sleep apnea syndrome

## Abstract

**Purpose:**

To investigate the real-world status of continuous positive airway pressure (CPAP) persistence in patients with sleep apnea syndrome (SAS) using administrative claims data in Japan.

**Methods:**

We designed a retrospective longitudinal study using administrative claims and medical check-up data collected from April 2014 to February 2022. We identified patients newly starting CPAP therapy as their first-line SAS treatment, and assessed patient characteristics, CPAP persistence rates, predictors of CPAP discontinuation, and second-line therapy options.

**Results:**

The analysis population (*n* = 13,007) was 76.2% male, and the mean age (± standard deviation) was 64.0 ± 15.0 years. CPAP persistence rates were 90.1% at 90 days, 77.1% at one year, 70.3% at two years, and 66.5% at three years. More discontinuation was noted in patients aged 18 to 44 (adjusted hazard ratio [95% confidence interval]: 1.27 [1.13–1.42]) and ≥ 65 (65–74 years, 1.18 [1.07–1.30]; ≥75 years, 1.59 [1.43–1.76]) than in those aged 45 to 64. Comorbidities of dementia (1.47 [1.25–1.73]), insomnia (1.26 [1.16–1.36]), and restless legs syndrome (RLS, 1.60 [1.16–2.23]) were also associated with more discontinuation. Testing with a Type 3 portable monitor (1.31 [1.21–1.42]), or no record of sleep testing before therapy (1.21 [1.09–1.36]), tended to be associated with more discontinuation than polysomnography. CPAP therapy starting in 2020 (0.72 [0.63–0.82]) and 2021 (0.63 [0.46–0.85]) resulted in better persistence than that starting in 2016. Only 6.1% of patients received second-line therapy after CPAP discontinuation.

**Conclusions:**

CPAP therapy was continued for at least a year by about 80% of patients. Because second-line therapies are rarely implemented, efforts are needed to ensure CPAP persistence by considering the factors that influence it. It is also crucial to raise awareness of alternative therapies.

**Registration number:**

NA.

**Supplementary Information:**

The online version contains supplementary material available at 10.1007/s11325-025-03417-w.

## Introduction

Sleep apnea syndrome (SAS) is a condition in which the upper airway is repeatedly obstructed during sleep, resulting in sleep fragmentation and intermittent hypoxia. SAS is a known risk factor for cardiovascular death, coronary artery disease, hypertension, atrial fibrillation, chronic heart failure, and stroke [1]. Obstructive sleep apnea (OSA), which accounts for the majority of sleep apnea, affects an estimated 936 million people worldwide [2].

Some patients with OSA experience symptoms such as excessive daytime sleepiness, while others do not, and comorbidities such as obesity vary from patient to patient [3]. The many combinations of OSA phenotypes necessitate, in turn, a variety of treatment needs and goals [4]. Due to differences in treatment efficacy among those phenotypes, treatment strategies tailored to the four categories of the Baveno classification have been proposed based on patients’ symptoms and comorbidities [5].

Continuous positive airway pressure (CPAP) is the gold standard for OSA treatment [1], and continued CPAP therapy is required to control apnea [6]. There are two major sources for evaluating CPAP adherence or persistence: telemonitoring and insurance claims data. Telemonitoring is well-suited to assessing adherence, as it enables measurement of the actual daily duration of CPAP use as well as the number of days of use. However, the diversity of CPAP device models and the lack of standardized data collection methods pose challenges [7]. In addition, telemonitoring data are not centrally managed in Japan, so it can be difficult to comprehensively assess adherence among patients undergoing CPAP therapy. Insurance claims data do not provide detailed information on adherence, but they can be used effectively to assess persistence, identified as the period from CPAP initiation to discontinuation. CPAP persistence rates have been widely studied in Western countries [8, 9] but only a few such studies have been reported from Asian countries including Japan [10–13].

The duration of CPAP persistence has been reported to vary by OSA phenotype [14, 15], which means that OSA phenotypes could be predictors of discontinuation of CPAP therapy. Previous studies have identified female sex, insomnia, chronic obstructive pulmonary disease (COPD), diabetes, and locomotive syndrome as predictors of discontinuation [8, 11, 16]. However, although these and other comorbidities have been associated with SAS [1, 6], to our knowledge no studies have comprehensively examined and reported their predictive potential for CPAP discontinuation. Furthermore, few studies have focused on the use of second-line therapy after discontinuation of CPAP therapy [8].

In this study, we used Japanese real-world data to investigate CPAP persistence rates in SAS patients and to explore predictors of CPAP discontinuation.

## Methods

### Study design and setting

This paper reports a retrospective longitudinal study using Japanese administrative claims and medical check-up data from April 2014 to February 2022, provided by the DeSC database (DeSC Healthcare Inc., Tokyo, Japan) [17]. That database contains anonymized processed information based on claims data for people insured by the society-managed employment-based health insurance association for individuals working at large companies and their families; the national health insurance for persons aged < 75 and not covered by another public health system; and the latter-stage elderly healthcare system for persons aged ≥ 75 years, and it also includes medical check-up data from some of the insurers. Patient information in the database was traceable across multiple hospitals and other medical facilities, as long as the patient remained a policy holder with the same health insurance program. The study was registered through the University Hospital Medical Information Network (UMIN) Clinical Trials Registry (UMIN000051874). The index date was defined as the date when SAS treatment was newly started, and the look-back period as the year just before the index date. The follow-up period was defined as up to three years after the index date or until withdrawal from insurance.

### Participants

Inclusion criteria required patients to be aged ≥ 18 years and to have newly initiated SAS treatment under a diagnosis of SAS (International Classification of Diseases 10th Revision [ICD-10] code: G473) between April 2015 and February 2021. SAS treatment was defined as therapies corresponding to the use codes listed in Supplemental Table S1, and the analysis population was defined as SAS patients who initiated CPAP therapy alone on the index date. Additional inclusion criteria included continuous enrollment in the database during the look-back period. To determine whether patients had received oral appliance (OA) therapy, which is one of the SAS treatment options, patients were required to be enrolled in insurance plans for which use of dental claims data was permitted. Patients were excluded who had received SAS treatment during the look-back period or who initiated a form of SAS treatment other than CPAP.

### Study measures and definitions

We evaluated patient characteristics and investigated CPAP persistence rate, predictors of CPAP discontinuation, and second-line therapy options after CPAP discontinuation. Changes in body weight before and after the start of CPAP therapy were also evaluated in some patients in the analysis population.

The applicable use codes for sleep tests are shown in Supplemental Table S2. Comorbidities were selected with reference to the international consensus statement on OSA [1] and the Japanese SAS clinical practice guidelines [6]. The corresponding use codes are shown in Supplemental Table S3.

### Statistical methods

Categorical variables were summarized by percentages and frequencies, and continuous variables by means and standard deviations (SDs) or medians and interquartile ranges if their distributions were skewed.

For CPAP persistence, we used the Kaplan-Meier method to estimate the cumulative persistence rate and median time to discontinuation. The time to discontinuation was defined as the period from the index date to the end date of CPAP therapy. Treatment was considered to be ongoing if the interval between CPAP claims was less than 180 days (the gap period). The end date for time to CPAP discontinuation was defined as the earliest of the following: (1) the date obtained by adding 30 days (grace period) to the last claim date for CPAP therapy during continuous treatment (defined as the discontinuation date, or the censored date if the actual discontinuation date would exceed the end of the observation period month); (2) the day before a new claim date for non-invasive positive pressure ventilation (NPPV) therapy or adaptive servo-ventilation (ASV) therapy (defined as the discontinuation date); or (3) the end of the observation period month (defined as the censored date). We also conducted a sensitivity analysis by altering the definition of time to discontinuation. For details, see Supplemental Methods S1. Subgroup analyses were conducted for age and type of sleep test.

To evaluate factors associated with discontinuation, we performed univariable and multivariable Cox regression analyses to estimate hazard ratios (HRs). This model included sex, age, number of oral concomitant medications, index year (the year when SAS treatment was newly started), number of beds in the medical facility, type of sleep test, and comorbidities as explanatory variables. Furthermore, we investigated factors associated with early and late discontinuation, setting 90 days after the index date as the cut-off. For this purpose, we first conducted Cox multivariable regression analysis using data from index date to the cut-off. Then we analyzed that data using the same model, conditioned on non-discontinuation and non-censoring at the cut-off.

For patients who discontinued CPAP and were available for 180-day follow-up after the last CPAP claims date, we evaluated second-line therapy that was started within 180 days after the last claims date. In detail, the proportion of patients who started second-line therapy and the type of SAS treatment were calculated. If multiple treatments were included during that period, the treatment claim closest to the last claims date was counted. Same analyzes were conducted for early and late discontinuation.

In patients for whom medical check-up data were available, changes in weight before and after the index date were analyzed for obese patients (BMI ≥ 25 kg/m^2^ [18] before the index date) and non-obese patients, respectively. This analysis was performed separately for patients who discontinued CPAP and other patients. Details of the analysis methods are shown in Supplemental Methods S2.

All statistical analyses were performed using SAS version 9.4. (SAS Institute; Cary, NC, USA).

## Results

### Study population

**Fig. 1:**
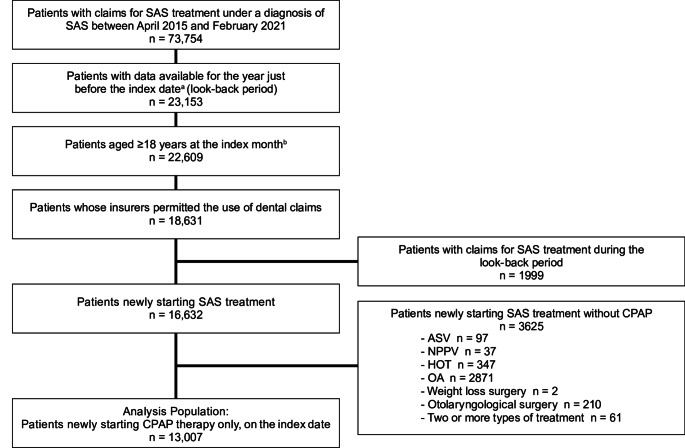
Flow diagram of enrolled patients. *SAS*, sleep apnea syndrome; *CPAP*, continuous positive airway pressure; *ASV*, adaptive servo-ventilation; *NPPV*, noninvasive positive pressure ventilation; *HOT*, home oxygen therapy; *OA*, oral appliances. ^**a**^Date when SAS treatment was started for the first time ^**b**^Month when SAS treatment was started for the first time

As shown in Fig. 1, we identified 16,632 patients newly starting SAS treatment between 2015 and 2021. Within that group, 13,007 (78.2%) started CPAP and 2871 (17.3%) began using OA. Other interventions, including ASV, NPPV, and home oxygen therapy (HOT), were only rarely prescribed. Patients who started CPAP therapy at the index date were included in the analysis population.

### Patient characteristics

Patient characteristics of the analysis population are shown in Table 1: 76.2% were male, and mean age ± SD was 64.0 ± 15.0 years. The most common comorbidity was hypertension (65.3%), followed by dyslipidemia (53.9%) and allergic rhinitis (44.5%). Sleep tests had been conducted within the one-year look-back period in 86.5% of patients, including type 3 portable monitor (type 3 PM) tests (58.3%) and polysomnography (PSG) (41.7%).

**Table 1:** Patient characteristics of analysis population

	CPAP
	*N* = 13,007
Sex, n (%)	
Male	9917 (76.2)
Female	3090 (23.8)
Age (years), mean ± SD	64.0 ± 15.0
Age category (years), n (%)	
18–44	1603 (12.3)
45–64	4158 (32.0)
65–74	3262 (25.1)
≥ 75	3984 (30.6)
Comorbidities, n (%)	
Hypertension	8492 (65.3)
Coronary artery disease	3199 (24.6)
Atrial fibrillation and atrial flutter	1606 (12.3)
Congestive heart failure	3504 (26.9)
Cerebrovascular disease	3012 (23.2)
Pulmonary hypertension	34 (0.3)
COPD	1533 (11.8)
Asthma	2508 (19.3)
Allergic rhinitis	5793 (44.5)
Chronic sinusitis	2090 (16.1)
Diabetes	5090 (39.1)
Gastroesophageal reflux disease	4981 (38.3)
Dyslipidemia	7017 (53.9)
NAFLD / NASH	1300 (10.0)
Renal disease	1003 (7.7)
Gout / Hyperuricemia	3008 (23.1)
Dementia	442 (3.4)
Anxiety disorder	1092 (8.4)
Depression	1441 (11.1)
Cancer	1362 (10.5)
Osteoporosis	1773 (13.6)
Fracture	957 (7.4)
Osteoarthritis	2989 (23.0)
Rheumatoid arthritis	407 (3.1)
Insomnia	3848 (29.6)
Narcolepsy	15 (0.1)
RLS	93 (0.7)
Glaucoma	1371 (10.5)
No. of oral concomitant medications, median (IQR)	4.0 (1.0, 7.0)
No. of oral concomitant medications, n (%)	
0 (No concomitant medications)	2477 (19.0)
1–4 (No polypharmacy)	4871 (37.4)
5–9 (Polypharmacy)	4198 (32.3)
≥ 10 (Excessive polypharmacy)	1461 (11.2)
Index year, n (%)	
2015	276 (2.1)
2016	1322 (10.2)
2017	1912 (14.7)
2018	2293 (17.6)
2019	3364 (25.9)
2020	3310 (25.4)
2021	530 (4.1)
Type of insurance, n (%)	
SHI	3243 (24.9)
NHI	5560 (42.7)
LSEHS	4204 (32.3)
No. of beds in medical facility^a^, n (%)	
≤ 19	7152 (55.0)
20–199	1906 (14.7)
200–399	1445 (11.1)
≥ 400	2473 (19.0)
Missing	31 (0.2)
Sleep test conducted, n (%)	
No	1756 (13.5)
Yes	11,251 (86.5)
Type of sleep test ^b^ conducted, n (%)	
Type 3 PM	6561 (58.3)
PSG	4690 (41.7)

*CPAP*, continuous positive airway pressure; *SD*, standard deviation; *COPD*, chronic obstructive pulmonary disease; *NAFLD*, nonalcoholic fatty liver disease; *NASH*, nonalcoholic steatohepatitis; *RLS*, restless legs syndrome; *IQR*, interquartile range; *SHI*, society-managed employment-based health insurance association; *NHI*, national health insurance; *LSEHS*, latter-stage elderly health care system; *Type 3 PM*, type 3 portable monitor; *PSG*, polysomnography

^a^Double aggregation is allowed

^b^The test closest to the index date was counted if multiple tests were conducted. PSG was counted if multiple tests were conducted on the same day

### CPAP persistence

**Fig. 2:**
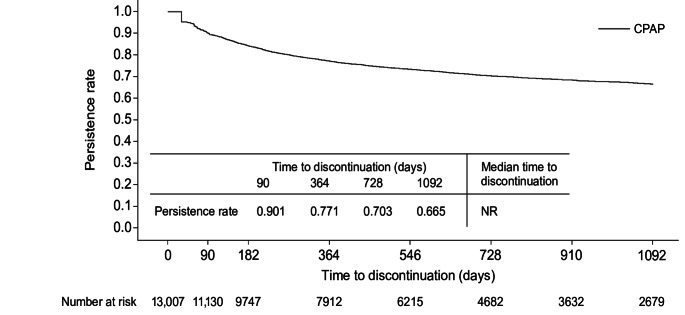
CPAP persistence: analysis using Kaplan-Meier method. *CPAP*, continuous positive airway pressure; *NR*, not reached

**Fig. 3:**
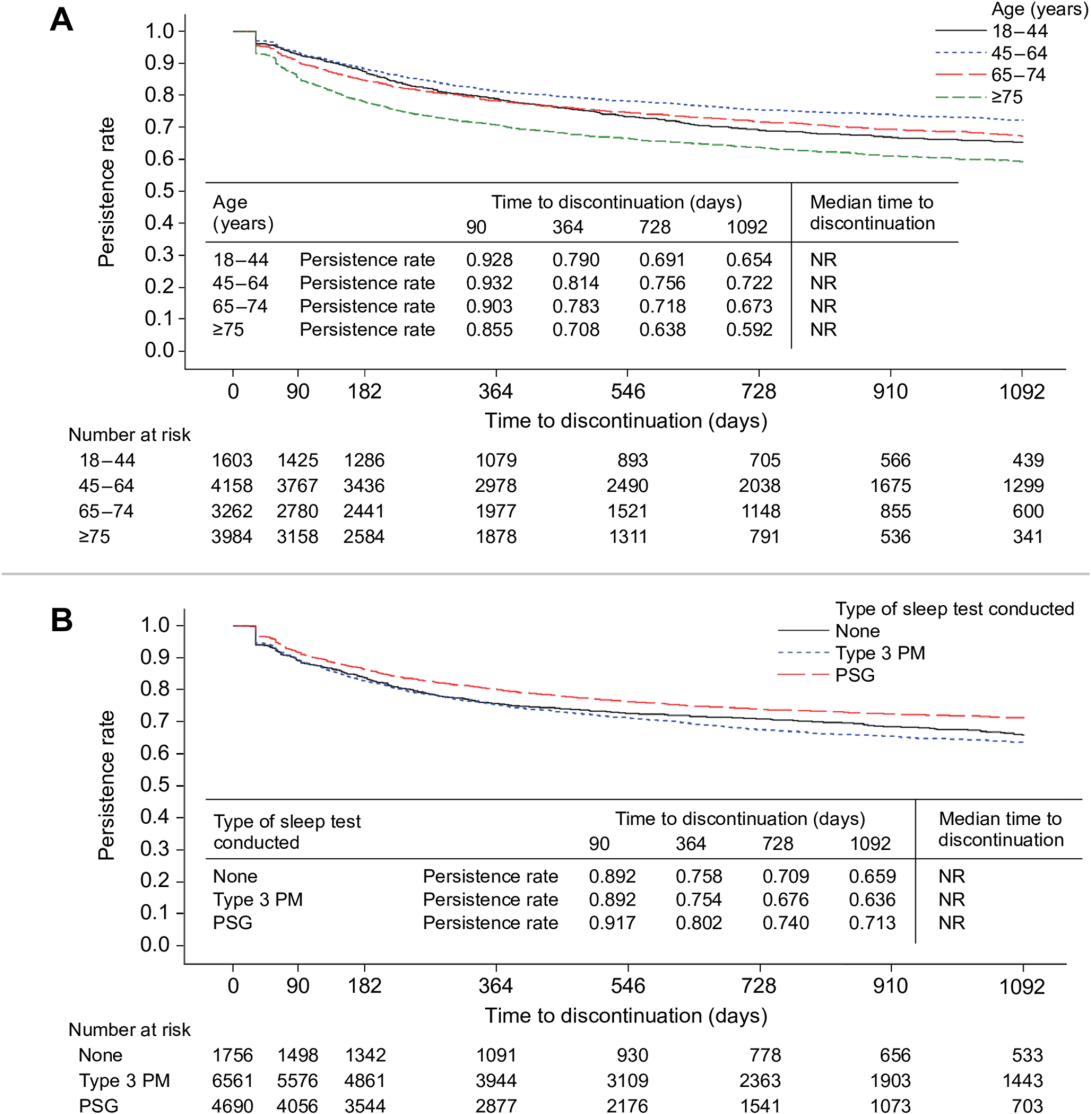
CPAP persistence: subgroup analysis using Kaplan-Meier method. **A** Subgroup analysis by age. **B** Subgroup analysis by type of sleep test conducted. *CPAP*, continuous positive airway pressure; *NR*, not reached; *Type 3 PM*, type 3 portable monitor; *PSG*, polysomnography

The median duration (interquartile range) of follow-up for persistence was 511.0 days (181.0–976.0). The CPAP persistence rate was 90.1% at 90 days, 77.1% at one year, 70.3% at two years, and 66.5% at three years (Fig. 2). Median time to discontinuation was not reached. Similar results were obtained for sensitivity analysis with a different definition of CPAP persistence (Supplemental Figure S1). Subgroup analyses by age and by type of sleep test are shown in Fig. 3.

### Predictors of CPAP discontinuation

**Fig. 4:**
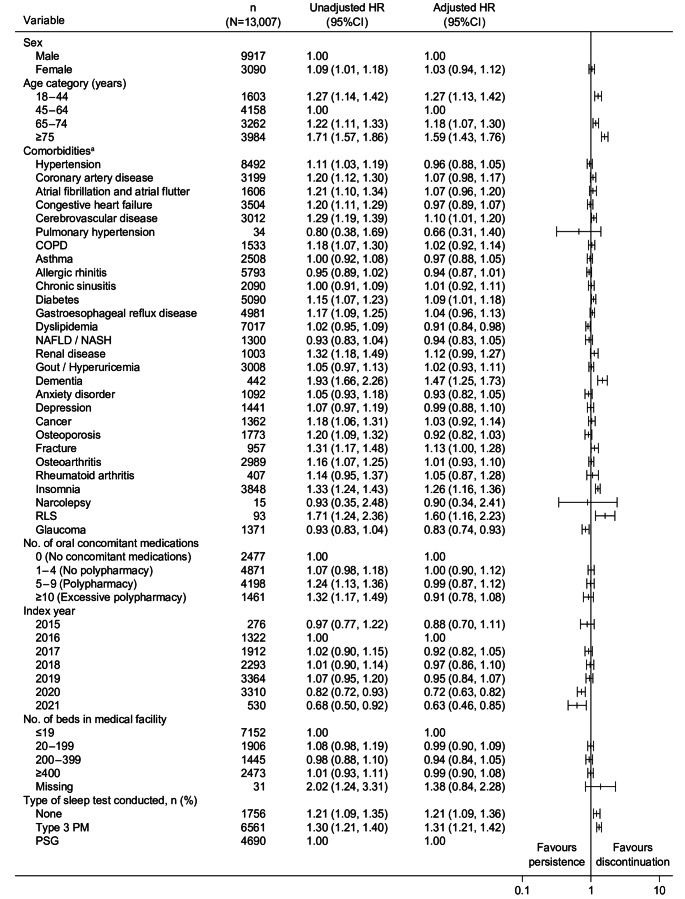
Predictors of CPAP discontinuation analyzed using Cox proportional hazards model. *CPAP*, continuous positive airway pressure; *HR*, hazard ratio; *CI*, confidence interval; *COPD*, chronic obstructive pulmonary disease; *NAFLD*, nonalcoholic fatty liver disease; *NASH*, nonalcoholic steatohepatitis; *RLS*, restless legs syndrome; *Type 3 PM*, type 3 portable monitor; *PSG*, polysomnography. ^a^Unadjusted/adjusted HR for each comorbidity is calculated using for reference a group of patients in which that comorbidity is not present

Predictors of CPAP discontinuation were analyzed using a Cox proportional hazards model (Fig. 4). Compared with 45–64 years of age, more discontinuation was noted at 18–44 years (adjusted hazard ratio [95% confidence interval]: 1.27 [1.13–1.42]), 65–74 years (1.18 [1.07–1.30]), and ≥ 75 years (1.59 [1.43–1.76]). Comorbidities that were associated with more discontinuation were cerebrovascular disease (1.10 [1.01–1.20]), diabetes (1.09 [1.01–1.18]), dementia (1.47 [1.25–1.73]), insomnia (1.26 [1.16–1.36]), and restless legs syndrome (RLS, 1.60 [1.16–2.23]). Better persistence was associated with dyslipidemia (0.91 [0.84–0.98]) and glaucoma (0.83 [0.74–0.93]). Compared with patients who had a PSG test, more discontinuation tended to be associated with those who had only a type 3 PM test (1.31 [1.21–1.42]) or no sleep test (1.21 [1.09–1.36]) during the year prior to CPAP initiation. Therapy that was started in 2020 (0.72 [0.63–0.82]) and 2021 (0.63 [0.46–0.85]) resulted in better treatment persistence than therapy started in 2016.

Because the Kaplan-Meier curves crossed for the 18–44 and 65–74 age groups (Fig. 3A), we focused on age-specific results when analyzing data by time of CPAP discontinuation. Early discontinuation was more common among patients aged 65–74 and ≥ 75 than those aged 45–64. Late discontinuation was more likely in those aged 18–44 and ≥ 75 than those aged 45–64 (Supplemental Figure S2).

### Second-line therapy after CPAP discontinuation

A total of 3534 patients discontinued CPAP therapy during the study period: 1262 patients (35.7%) within the first 3 months (early discontinuation), and 2272 patients (64.3%) after that point (late discontinuation). Among these 3534 patients, 214 (6.1%) subsequently received some other form of SAS treatment: the most common was OA therapy in 122 patients (57.0%), followed by ASV therapy in 41 (19.2%), otolaryngological surgery in 20 (9.3%), and HOT in 17 (7.9%) (Supplemental Table S4).

### Changes in body weight

We assessed changes in weight before and after the start of CPAP therapy (Supplemental Table S5). Within the analysis population, weight and BMI data at medical check-up were available for 2965 patients, of whom 1796 (60.6%) were obese before therapy was started. Among the patients who discontinued CPAP (*n* = 275), only 5.1% had weight loss of ≥ 10% [19].

## Discussion

In this study using Japanese administrative claims data, we investigated CPAP persistence and predictors of discontinuation in patients with SAS who newly started treatment.

In our study, the CPAP persistence rate was 77.1% at one year, 70.3% at two years, and 66.5% at three years. The one-year CPAP persistence rate in large-scale epidemiological studies was 81.1% in the U.S. [20] and 76.9% in France [8], similar to findings in the present study. Other Japanese findings have been obtained from single-center reports that show a CPAP persistence rate of 92.8% at three years [11] and 89.8% at five years [12], and a multicenter study that reported 84.9% at one year [13]. Those higher percentages could be attributed to the studies being conducted at specialized sleep medicine facilities, where higher persistence rates would be expected. In contrast, the present study is based on insurance claims data obtained from a variety of medical institutions, and can be expected to reflect the real-world treatment situation in Japan.

Next, we explored potential predictors of CPAP discontinuation. Our results suggested an association with the factors of age, comorbidities, sleep test, and index year. Previous reports have described a U-shaped relationship between age and CPAP discontinuation [8, 9], which was confirmed by the present study. Younger patients tended to discontinue CPAP therapy later than other age groups, while older patients tended to discontinue earlier, consistent with trends reported previously [9]. This indicates that, in addition to follow-up care for the older age group, it is important to provide medical care that encourages younger patients to persist with treatment, especially beyond the first 3 months.

Dementia [21] and insomnia [22] have been reported as predictors of CPAP discontinuation. Recently the concept of “comorbid insomnia and sleep apnea (COMISA)” has been introduced [23], insomnia being associated with a particularly high proportion of comorbidity of approximately 30% (Table 1). Patients with COMISA can experience lower CPAP adherence [24] as well as negative impacts on cardiovascular disorders [25] and overall prognosis [26]. To our knowledge, RLS and glaucoma have not been reported as predictors of CPAP discontinuation. Perhaps sleep was disturbed by the lower extremity discomfort that appears at night in RLS patients, which could disturb CPAP device placement and gradually increase CPAP discontinuation. Although there have been reports indicating the therapeutic benefits of CPAP for glaucoma [27], the underlying reasons remain unexplained for the better persistence with CPAP therapy among glaucoma patients in this study. Statistically significant differences have been noted in patients with cerebrovascular disease, diabetes, and dyslipidemia, but the HR range was only 0.91 to 1.10. Further study will be required to determine the clinical significance of these differences, including whether they are based in the disease itself or are due to factors such as patient characteristics that are disease-specific.

In comparison with patients who had a PSG test, the HR for CPAP discontinuation was 1.31 in patients who had only a type 3 PM test and 1.21 in those who had no sleep assessment at all during the year before starting CPAP therapy. This could be because medical institutions that perform PSG tests have greater expertise in sleep medicine and can provide more appropriate patient management. Of note, no differences in short-term CPAP adherence have been reported between the patients diagnosed using home sleep apnea testing and those who underwent PSG testing [28, 29]. There are no studies comparing CPAP adherence between the two tests over the long term; future studies are warranted. In addition, of the SAS patients who newly started CPAP therapy in the present study, approximately 14% had not participated in a sleep test during the year before therapy was initiated. It is possible, however, that patients who discontinued CPAP therapy in the past may have resumed treatment, or that some of those patients started CPAP therapy based on the results of sleep tests that were performed more than one year previously, and the reasons for this situation were not identified under the current study design.

Interestingly, the CPAP persistence rate was better for therapy started in 2020 and 2021 than for other years. A study showed improved adherence to CPAP therapy in SAS patients during the Coronavirus disease 2019 (COVID-19) lockdown than before that period [30]. This may have been because patients hoped that CPAP therapy would prevent the worsening of COVID-19 due to complications of SAS [30].

Findings from this study suggest that few patients discontinued treatment because of weight loss. Instead, most discontinuations appear attributable to difficulties in tolerating CPAP therapy. This is because, in subgroup analysis among the patients who discontinued CPAP, only about 5% of obese patients lost 10% or more of their body weight (Supplemental Table S5).

In addition, of the patients who discontinued CPAP therapy, 93.9% received no second-line therapy within at least the first six months after discontinuation (Supplemental Table S4). These findings suggest that real-world clinical settings include a large number of patients with moderate or severe SAS who remain untreated or insufficiently treated. A meta-analysis of randomized clinical trials (RCTs) in SAS patients with coronary artery disease or stroke has shown no mortality-related benefits of CPAP therapy [31]. However, the results of this meta-analysis were also influenced by the fact that the RCTs excluded patients who had symptoms of excessive daytime sleepiness, included patients with low CPAP adherence, and had only a small number of death events [32]. Conversely, in addition to the results of earlier observational studies [31], recent observational studies [32, 33] have shown that persistence or resumption of CPAP therapy reduces mortality in SAS patients. Therefore, for a large number of SAS patients in a real-world clinical setting, CPAP persistence should be aggressively encouraged with the expectation that such persistence may reduce mortality. The predictors that were identified in this study appear to encourage approaches that are well-suited to younger as well as older patients, care that takes comorbidities into account, encouragement for patients to try medical facilities specializing in sleep medicine, and patient education to ensure the rate of CPAP persistence.

This study has several limitations. First, it is a secondary-use study of administrative claims data and medical check-up data, so the definitions of disease and outcome are of only limited validity. However, the results of sensitivity analyses for CPAP persistence were similar to those in the primary analysis, confirming the robustness of our results (Supplemental Figure S1). Second, this study targeted SAS patients receiving CPAP therapy and excluded patients who had mild SAS disease or who did not want to be treated. In addition, the study included data from a large number of older patients, so our findings may not accurately represent the overall CPAP persistence rate. However, we believe that the actual situation is reflected in our findings for CPAP persistence rate by age group. Third, our study does not provide information on the severity of SAS as determined by assessment criteria such as the Apnea-Hypopnea Index or the intensity of subjective symptoms such as daytime sleepiness, so we were unable to evaluate potential relationships between CPAP persistence rate and severity or subjective symptom intensity. Fourth, the insurance claims data provided no measure of CPAP adherence, so it is unclear to what extent the patients actually used CPAP. Fifth, other risk factors for discontinuation, including functional aspects of OSA, endotypes, and other phenotypes, were also not captured in the insurance claims data and thus could not be assessed, so their impact could not be considered here.

## Conclusion

We investigated the status of CPAP persistence and predictors of CPAP discontinuation in Japanese patients with SAS. At one year, CPAP persistence rate was slightly less than 80%. Very few patients received second-line therapy after CPAP discontinuation. CPAP discontinuation was relatively high in young and older patients, in those with dementia, insomnia, and RLS, and in those who received only a type 3 PM test before starting CPAP therapy. Alternative treatments to CPAP are limited at present, so efforts should be focused on the factors affecting CPAP discontinuation. Increasing awareness of alternative therapies is also crucial, especially for patients who discontinue CPAP.

## Electronic supplementary material

Below is the link to the electronic supplementary material.

Supplementary Material 1

## Data Availability

The datasets generated and/or analyzed during the current study were obtained from data accessed under license with DeSC Healthcare Inc. (Tokyo, Japan) and are not publicly available due to restrictions placed by DeSC Healthcare Inc. on the use of that data.
