# Supplementary material for: Real-world status of continuous positive airway pressure (CPAP) persistence in patients with sleep apnea syndrome (SAS): a retrospective longitudinal study of administrative claims data in Japan
Source: Sleep Breath. 2025 Jul 17;29(4):245. doi: 10.1007/s11325-025-03417-w (PMC12271271; doi:10.1007/s11325-025-03417-w)
Supplement: Supplementary file 1 — Supplementary Material 1 [file 11325_2025_3417_MOESM1_ESM.pdf]

Supplemental Material

Sleep and Breathing

Real-word status of continuous positive airway pressure (CPAP) persistence in patients with sleep apnea syndrome (SAS): A retrospective longitudinal study of administrative claims data in Japan

Hiroyuki Takahashi[1], Shiori Yoshida[2], Akihiro Nakajima[2], Ruriko Koto[1], Hideaki Nakayama[3]

1. Medical Science Department, Teijin Pharma Limited, Tokyo, Japan
2. Clinical Development Control Department, Teijin Pharma Limited, Tokyo, Japan
3. Department of Somnology, Tokyo Medical University Hospital, Tokyo, Japan

Contents:

Table S1: Definition of SAS treatment — 2

Table S2: Definition of sleep tests — 3

Table S3: Definition of number of concomitant oral medications and comorbidity — 4

Table S4: Second-line therapy after discontinuation from CPAP therapy — 6

Table S5: Weight change before and after starting CPAP therapy — 7

Figure S1: CPAP persistence: sensitivity analysis using Kaplan-Meier method — 8

Figure S2: Predictors of CPAP discontinuation analyzed using Cox proportional hazards model, stratified by discontinuation period (early or late) — 9

Methods S1: Definitions of sensitivity analysis and last claims date — 11

Methods S2: Analysis population and evaluation method for weight change before and after starting CPAP therapy — 12

Correspondence to:

Hiroyuki Takahashi hiroy.takahashi@teijin.co.jp

Table S1: Definition of SAS treatment

Any of the following procedure codes were recorded at the index date.

CPAP
114041310, 114010370

ASV
114041210

NPPV
114009610

HOT
114006210, 114004910

OA
309007150, 309007050

Weight loss surgery
150378010

Otolaryngological surgery
150100110, 150099010, 150097310, 150097450, 150356410, 150103310, 150104810, 150104910, 150114350

Jaw osteoplasty
150116710, 150116810, 150336110, 150268410, 150268510, 150268610, 150268710, 150336210, 150356710

SAS, sleep apnea syndrome; *CPAP*, continuous positive airway pressure; *ASV*, adaptive servo-ventilation; *NPPV*, noninvasive positive pressure ventilation; *HOT*, home oxygen therapy; *OA*, oral appliances

Table S2: Definition of sleep tests

Any of the following procedure codes were recorded during the look-back period[a].
Type 3 PM[b]<br>160119410, 160188750
PSG<br>160218510, 160218610, 160160510

Type 3 PM, type 3 portable monitor; *PSG*, polysomnography

[a] The test closest to the index date was counted if multiple tests were conducted. PSG was counted if multiple tests were conducted on the same day

[b] Out-of-center sleep test type 3 PM as defined by the American Academy of Sleep Medicine

Table S3: Definition of number of concomitant oral medications and comorbidity

Definition of the number of oral concomitant medications
Number of active ingredients in medicines for which the index date fell within the prescribing period. Oral medications that were prescribed for ≥7 days, excluding those with an ATC code of J (except for J05) and those prescribed on an as-needed basis.

Definition of comorbidity
Any of the following ICD-10 codes or claims codes were recorded during the look-back period.

Hypertension
ICD-10: I10, I11, I12, I13, I15

Coronary artery disease
ICD-10: I20, I21, I22, I23, I24, I25

Atrial fibrillation and atrial flutter
ICD-10: I48

Congestive heart failure
ICD-10: I099, I110, I130, I132, I255, I420, I425, I426, I427, I428, I429, I43, I50, P290

Cerebrovascular disease
ICD-10: G45, G46, H340, I60, I61, I62, I63, I64, I65, I66, I67, I68, I69

Pulmonary hypertension
ICD-10: I270, I272, I278, I279

COPD
ICD-10: J41, J42, J43, J44

Asthma
ICD-10: J45, J46

Allergic rhinitis
ICD-10: J30

Chronic sinusitis
ICD-10: J32

Diabetes
ICD-10: E10, E11, E12, E13, E14

Gastroesophageal reflux disease
ICD-10: K21

Dyslipidemia
ICD-10: E78

NAFLD / NASH
ICD-10: K758, K760

Renal disease
ICD-10: I120, I131, N032, N033, N034, N035, N036, N037, N052, N053, N054, N055, N056, N057, N18, N19, N250, Z490, Z491, Z492, Z940, Z992

Gout / Hyperuricemia
ICD-10: M10, E790

Dementia
ICD-10: F00, F01, F02, F03, F051, G30, G311

Table S3: Continued.

Anxiety disorder<br>ICD-10: F064, F40, F41, F930
Depression<br>ICD-10: F32, F33
Cancer<br>ICD-10: C00–C14, C15–C26, C30–C39, C40–C41, C43, C45–C49, C50, C51–C58, C60–C63, C64–C68, C69–C72, C73–C75, C76, C81, C82, C83, C84, C85, C88, C90, C91, C92, C93, C94, C95, C96, C97
Osteoporosis<br>ICD-10: M80, M81, M82
Fracture<br>ICD-10: S02, S12, S22, S32, S42, S52, S62, S72, S82, S92, T02, T08, T10, T12, T142
Osteoarthritis<br>ICD-10: M15, M16, M17, M18, M19
Rheumatoid arthritis<br>ICD-10: M05, M06
Insomnia<br>ICD-10: F510, G470
Narcolepsy<br>ICD-10: G474
RLS<br>Diagnosis codes: 8831218
Glaucoma<br>ICD-10: H401, H402, H403, H404, H405, H406, H408, H409, H42, Q150

ATC, anatomical therapeutic chemical; *ICD-10*, International Classification of Diseases 10th revision; *COPD*, chronic obstructive pulmonary disease; *NAFLD*, nonalcoholic fatty liver disease; *NASH*, nonalcoholic steatohepatitis; *RLS*, restless legs syndrome

Table S4: Second-line therapy after discontinuation from CPAP therapy

Second-line therapy for SAS, n (%)	Discontinuation from CPAP therapy		
	All<br>n = 3534	Early discontinuation[c]<br>n = 1262	Late discontinuation[d]<br>n = 2272
No[a]	3320 (93.9)	1164 (92.2)	2156 (94.9)
Yes[a]	214 (6.1)	98 (7.8)	116 (5.1)
ASV[b]	41 (19.2)	14 (14.3)	27 (23.3)
NPPV[b]	9 (4.2)	7 (7.1)	2 (1.7)
HOT[b]	17 (7.9)	12 (12.2)	5 (4.3)
OA[b]	122 (57.0)	58 (59.2)	64 (55.2)
Weight loss surgery[b]	0 (0.0)	0 (0.0)	0 (0.0)
Otolaryngological surgery[b]	20 (9.3)	5 (5.1)	15 (12.9)
Jaw osteoplasty[b]	0 (0.0)	0 (0.0)	0 (0.0)
Two or more types of treatment[b]	5 (2.3)	2 (2.0)	3 (2.6)

CPAP, continuous positive airway pressure; *SAS*, sleep apnea syndrome; *ASV*, adaptive servo-ventilation; *NPPV*, noninvasive positive pressure ventilation; *HOT*, home oxygen therapy; *OA*, oral appliances

[a] Percentage was calculated using the number of patients who discontinued from CPAP therapy as the denominator

[b] Percentage was calculated using the number of patients who received second-line therapy for SAS as the denominator

[c] Patients who discontinued <90 days after the index date

[d] Patients who did not discontinue or were not censored <90 days after the index date

Table S5: Weight change before and after starting CPAP therapy

	BMI before the index date (kg/m^2)[a]			
	<25		≥25	
	Discontinuation[b] n = 494	No discontinuation[c] n = 1460	Discontinuation[b] n = 694	No discontinuation[c] n = 2092
Weight and BMI data[d] available at the time of medical check-up, n (%)	148 (30.0)	1021 (69.9)	275 (39.6)	1521 (72.7)
Weight before the index date (kg), mean ± SD	63.62 ± 9.19	63.88 ± 8.28	82.70 ± 13.11	84.38 ± 14.37
Weight after the index date (kg), mean ± SD	63.93 ± 9.25	65.16 ± 8.84	81.90 ± 13.69	84.38 ± 14.25
Weight change (%), mean ± SD	0.61 ± 4.65	2.02 ± 4.47	−0.94 ± 5.86	0.13 ± 5.03
Weight change, n (%)				
≤−10%	3 (2.0)	8 (0.8)	14 (5.1)	50 (3.3)
≤−20%	0 (0.0)	0 (0.0)	4 (1.5)	0 (0.0)
≤−30%	0 (0.0)	0 (0.0)	1 (0.4)	0 (0.0)

CPAP, continuous positive airway pressure; *BMI*, body mass index; *SD*, standard deviation

[a] Patients with records of BMI value before the index date

[b] The data closest to the last claims date are used for patients who persisted with treatment for 180 days or more after the index date and for whom medical check-up data are available after the last claims date, and if data are a vailable for two or more points

[c] The data closest to the last claims date are used for patients who persisted with treatment for 180 days or more after the index date and for whom medical check-up data are available during the treatment period, and if data are available for two or more points

[d] Medical check-up before and after the index date

Figure S1: CPAP persistence: sensitivity analysis using Kaplan-Meier method

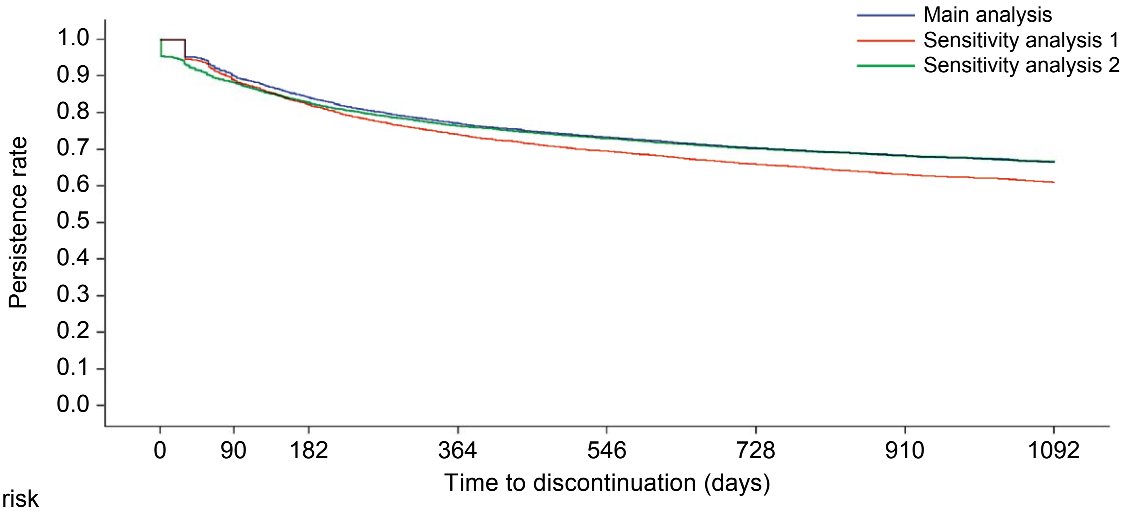

Number at risk								
Main analysis	13,007	11,130	9747	7912	6215	4682	3632	2679
Sensitivity analysis 1	13,007	11,045	9588	7681	5977	4459	3427	2526
Sensitivity analysis 2	13,007	10,737	9454	7675	6032	4554	3533	2604

Type of analysis	Gap period / grace period (days)		Time to discontinuation (days) 90	364	728	1092	Median time to discontinuation
Main analysis	180 / 30	Persistence rate	0.901	0.771	0.703	0.665	NR
Sensitivity analysis 1	120 / 30	Persistence rate	0.890	0.741	0.659	0.610	NR
Sensitivity analysis 2	180 / 0	Persistence rate	0.883	0.764	0.703	0.667	NR

CPAP, continuous positive airway pressure; *NR*, not reached

Figure S2: Predictors of CPAP discontinuation analyzed using Cox proportional hazards model, stratified by discontinuation period (early or late)

(A) Early withdrawal

Variable	n (N=13,007)	Unadjusted HR (95%CI)	Adjusted HR (95%CI)
Sex			
Male	9917	1.00	1.00
Female	3090	1.16 (1.02, 1.32)	1.01 (0.88, 1.17)
Age category (years)			
18−44	1603	1.07 (0.86, 1.33)	1.05 (0.84, 1.32)
45−64	4158	1.00	1.00
65–74	3262	1.45 (1.24, 1.71)	1.44 (1.22, 1.71)
≥75	3984	2.24 (1.94, 2.59)	2.19 (1.83, 2.61)
Comorbidities[a]			
Hypertension	8492	1.20 (1.07, 1.35)	0.91 (0.79, 1.06)
Coronary artery disease	3199	1.30 (1.16, 1.47)	1.04 (0.90, 1.20)
Atrial fibrillation and atrial flutter	1606	1.47 (1.27, 1.70)	1.20 (1.01, 1.42)
Congestive heart failure	3504	1.40 (1.24, 1.57)	1.03 (0.89, 1.20)
Cerebrovascular disease	3012	1.39 (1.23, 1.56)	1.05 (0.92, 1.21)
Pulmonary hypertension	34	0.91 (0.29, 2.81)	0.64 (0.20, 2.01)
COPD	1533	1.15 (0.98, 1.36)	0.92 (0.77, 1.09)
Asthma	2508	1.00 (0.87, 1.15)	0.99 (0.85, 1.15)
Allergic rhinitis	5793	0.88 (0.78, 0.98)	0.86 (0.76, 0.97)
Chronic sinusitis	2090	1.01 (0.87, 1.17)	1.06 (0.90, 1.24)
Diabetes	5090	1.21 (1.09, 1.36)	1.09 (0.96, 1.23)
Gastroesophageal reflux disease	4981	1.22 (1.09, 1.36)	0.98 (0.86, 1.12)
Dyslipidemia	7017	1.09 (0.97, 1.22)	0.94 (0.82, 1.06)
NAFLD / NASH	1300	0.88 (0.72, 1.07)	0.90 (0.74, 1.09)
Renal disease	1003	1.41 (1.18, 1.69)	1.12 (0.92, 1.36)
Gout / Hyperuricemia	3008	1.04 (0.91, 1.18)	0.98 (0.85, 1.13)
Dementia	442	2.03 (1.62, 2.55)	1.34 (1.06, 1.71)
Anxiety disorder	1092	1.08 (0.89, 1.31)	0.95 (0.78, 1.16)
Depression	1441	1.02 (0.85, 1.21)	0.97 (0.81, 1.18)
Cancer	1362	1.24 (1.05, 1.47)	0.99 (0.83, 1.18)
Osteoporosis	1773	1.34 (1.16, 1.55)	0.89 (0.74, 1.06)
Fracture	957	1.54 (1.29, 1.85)	1.23 (1.02, 1.49)
Osteoarthritis	2989	1.33 (1.18, 1.51)	1.06 (0.93, 1.22)
Rheumatoid arthritis	407	1.38 (1.05, 1.81)	1.21 (0.91, 1.61)
Insomnia	3848	1.40 (1.25, 1.57)	1.26 (1.11, 1.44)
RLS	93	1.98 (1.23, 3.20)	1.88 (1.16, 3.05)
Glaucoma	1371	0.99 (0.82, 1.18)	0.83 (0.69, 0.99)
No. of oral concomitant medications			
0 (No concomitant medications)	2477	1.00	1.00
1−4 (No polypharmacy)	4871	1.14 (0.97, 1.35)	0.98 (0.82, 1.18)
5−9 (Polypharmacy)	4198	1.42 (1.20, 1.68)	0.97 (0.79, 1.20)
≥10 (Excessive polypharmacy)	1461	1.56 (1.27, 1.91)	0.92 (0.71, 1.20)
Index year			
2015	276	1.06 (0.71, 1.59)	0.89 (0.59, 1.34)
2016	1322	1.00	1.00
2017	1912	0.99 (0.80, 1.24)	0.85 (0.68, 1.07)
2018	2293	1.06 (0.86, 1.31)	0.96 (0.77, 1.19)
2019	3364	1.09 (0.89, 1.33)	0.86 (0.70, 1.06)
2020	3310	0.89 (0.72, 1.10)	0.68 (0.55, 0.85)
2021	530	0.63 (0.41, 0.96)	0.50 (0.33, 0.77)
No. of beds in medical facility			
≤19	7152	1.00	1.00
20−199	1906	1.07 (0.92, 1.26)	0.93 (0.79, 1.09)
200−399	1445	0.96 (0.80, 1.15)	0.89 (0.73, 1.07)
≥400	2473	1.01 (0.87, 1.17)	0.95 (0.82, 1.11)
Missing	31	3.54 (1.84, 6.84)	2.08 (1.06, 4.10)
Type of sleep test conducted, n (%)			
None	1756	1.34 (1.12, 1.59)	1.34 (1.12, 1.61)
Type 3 PM	6561	1.32 (1.16, 1.50)	1.32 (1.16, 1.50)
PSG	4690	1.00	1.00

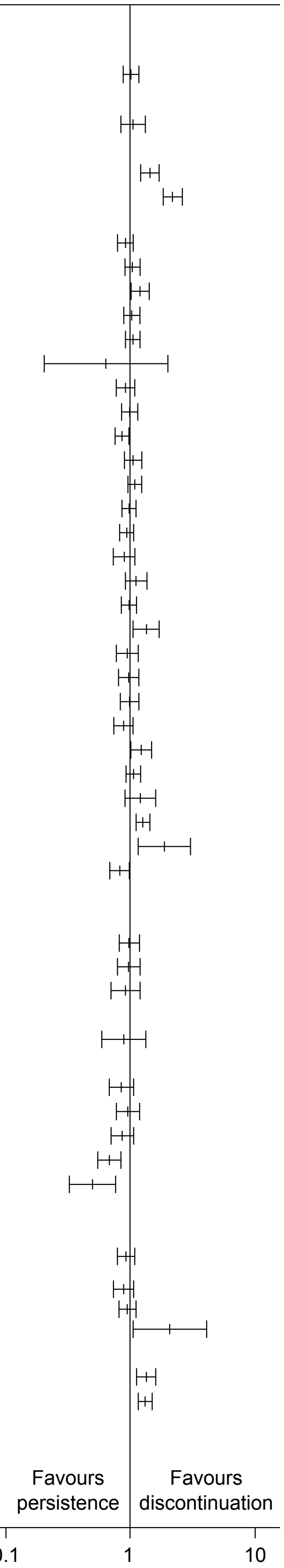

Figure S2: Continued.

(B) Late withdrawal

Variable	n (N=11,113)	Unadjusted HR (95%CI)	Adjusted HR (95%CI)
Sex			
Male	8543	1.00	1.00
Female	2570	1.05 (0.95, 1.16)	1.03 (0.92, 1.15)
Age category (years)			
18−44	1425	1.35 (1.19, 1.54)	1.36 (1.19, 1.55)
45−64	3762	1.00	1.00
65–74	2776	1.12 (1.00, 1.25)	1.07 (0.95, 1.21)
≥75	3150	1.46 (1.31, 1.62)	1.32 (1.16, 1.51)
Comorbidities[a]			
Hypertension	7160	1.06 (0.97, 1.15)	0.99 (0.89, 1.10)
Coronary artery disease	2649	1.15 (1.04, 1.26)	1.09 (0.97, 1.22)
Atrial fibrillation and atrial flutter	1279	1.05 (0.92, 1.20)	0.98 (0.84, 1.14)
Congestive heart failure	2855	1.09 (0.99, 1.20)	0.93 (0.83, 1.05)
Cerebrovascular disease	2447	1.23 (1.12, 1.35)	1.13 (1.01, 1.26)
Pulmonary hypertension	30	0.74 (0.28, 1.98)	0.69 (0.26, 1.85)
COPD	1279	1.19 (1.05, 1.35)	1.10 (0.96, 1.25)
Asthma	2145	0.99 (0.89, 1.10)	0.95 (0.85, 1.06)
Allergic rhinitis	4983	1.00 (0.92, 1.09)	0.99 (0.90, 1.08)
Chronic sinusitis	1795	1.00 (0.89, 1.12)	0.99 (0.88, 1.11)
Diabetes	4260	1.11 (1.02, 1.21)	1.09 (0.99, 1.20)
Gastroesophageal reflux disease	4144	1.14 (1.05, 1.24)	1.08 (0.98, 1.19)
Dyslipidemia	5947	0.98 (0.90, 1.06)	0.89 (0.81, 0.98)
NAFLD / NASH	1122	0.96 (0.84, 1.11)	0.96 (0.83, 1.11)
Renal disease	799	1.26 (1.09, 1.47)	1.13 (0.96, 1.33)
Gout / Hyperuricemia	2554	1.05 (0.96, 1.16)	1.04 (0.93, 1.15)
Dementia	325	1.86 (1.50, 2.29)	1.54 (1.23, 1.92)
Anxiety disorder	922	1.03 (0.89, 1.19)	0.91 (0.78, 1.07)
Depression	1240	1.11 (0.97, 1.26)	0.99 (0.86, 1.14)
Cancer	1116	1.13 (0.99, 1.30)	1.05 (0.91, 1.21)
Osteoporosis	1423	1.12 (0.99, 1.26)	0.94 (0.81, 1.09)
Fracture	754	1.17 (1.00, 1.37)	1.06 (0.89, 1.25)
Osteoarthritis	2468	1.06 (0.96, 1.18)	0.98 (0.87, 1.09)
Rheumatoid arthritis	327	1.00 (0.77, 1.28)	0.95 (0.73, 1.23)
Insomnia	3190	1.29 (1.18, 1.41)	1.25 (1.13, 1.39)
Narcolepsy	15	1.47 (0.55, 3.90)	1.34 (0.50, 3.59)
RLS	70	1.53 (0.98, 2.37)	1.42 (0.91, 2.22)
Glaucoma	1170	0.89 (0.77, 1.03)	0.84 (0.72, 0.97)
No. of oral concomitant medications			
0 (No concomitant medications)	2204	1.00	1.00
1−4 (No polypharmacy)	4237	1.04 (0.93, 1.17)	1.01 (0.89, 1.15)
5−9 (Polypharmacy)	3483	1.15 (1.03, 1.30)	1.00 (0.85, 1.16)
≥10 (Excessive polypharmacy)	1189	1.20 (1.03, 1.40)	0.91 (0.74, 1.12)
Index year			
2015	246	0.94 (0.71, 1.23)	0.87 (0.66, 1.15)
2016	1160	1.00	1.00
2017	1686	1.03 (0.89, 1.19)	0.97 (0.83, 1.12)
2018	2005	0.99 (0.86, 1.15)	0.98 (0.84, 1.13)
2019	2943	1.06 (0.92, 1.22)	1.00 (0.86, 1.15)
2020	2889	0.77 (0.65, 0.90)	0.72 (0.61, 0.86)
2021	184	0.78 (0.50, 1.22)	0.78 (0.50, 1.21)
No. of beds in medical facility			
≤19	6124	1.00	1.00
20−199	1612	1.08 (0.96, 1.22)	1.03 (0.91, 1.17)
200−399	1244	0.99 (0.87, 1.14)	0.98 (0.85, 1.13)
≥400	2111	1.01 (0.91, 1.13)	1.01 (0.90, 1.13)
Missing	22	1.30 (0.62, 2.74)	0.99 (0.46, 2.10)
Type of sleep test conducted, n (%)			
None	1497	1.15 (1.01, 1.31)	1.15 (1.00, 1.32)
Type 3 PM	5565	1.29 (1.18, 1.42)	1.31 (1.19, 1.44)
PSG	4051	1.00	1.00

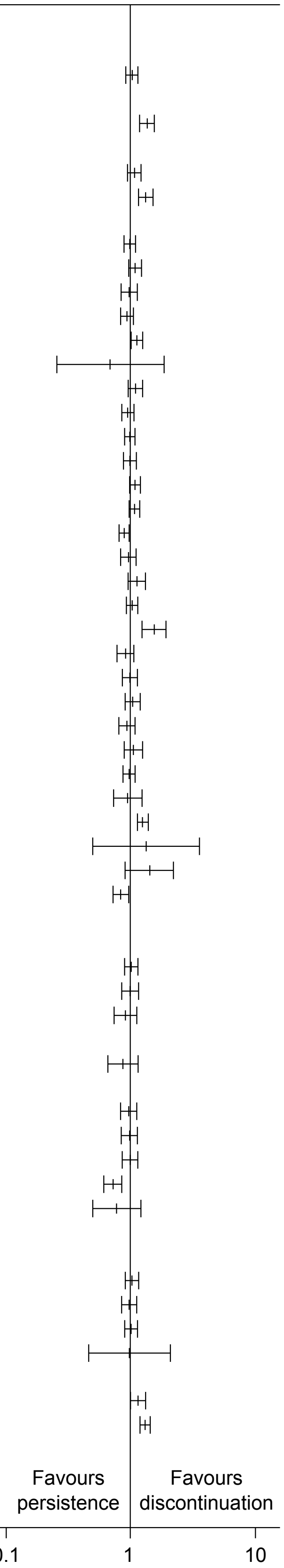

CPAP, continuous positive airway pressure; *HR*, hazard ratio; *CI*, confidence interval; *COPD*, chronic obstructive pulmonary disease; *NAFLD*, nonalcoholic fatty liver disease; *NASH,* nonalcoholic steato-hepatitis; *RLS*, restless legs syndrome; Type 3 PM, type 3 portable monitor; *PSG*, polysomnography

[a]Unadjusted/adjusted HR for each comorbidity is calculated using for reference a group of patients in which that comorbidity is not present

Methods S1: Definitions of sensitivity analysis and last claims date

Definition of sensitivity analysis

Sensitivity analysis 1: The gap period was changed from 180 days to 120 days.

Sensitivity analysis 2: The grace period was changed from 30 days to 0 days.

Definition of last claims date

1. The last claims date for CPAP therapy for patients whose end date for time to CPAP discontinuation is "day when 30 days (grace period) was added to the last claims date for CPAP therapy"
2. The end date of the time to CPAP discontinuation for patients whose end date is other than described above

CPAP, continuous positive airway pressure

Methods S2: Analysis population and evaluation method for weight change before and after starting CPAP therapy

---

Analysis population

Patients with weight and BMI data at the time of medical check-up that meet the following conditions are selected as the analysis population. Subgroup analysis by baseline BMI is conducted.

1. Before the index date (baseline)
   If data are available from the look-back period, those data are used. The data closest to the index date are used if data are available for two or more time points.
2. After the index date
   A) Patients who discontinued from CPAP therapy
      If patients persist with CPAP therapy for 180 days or more after the index date and data are available after the last claims date, those data are used. The data closest to the last claims date are used if data are available for two or more points.

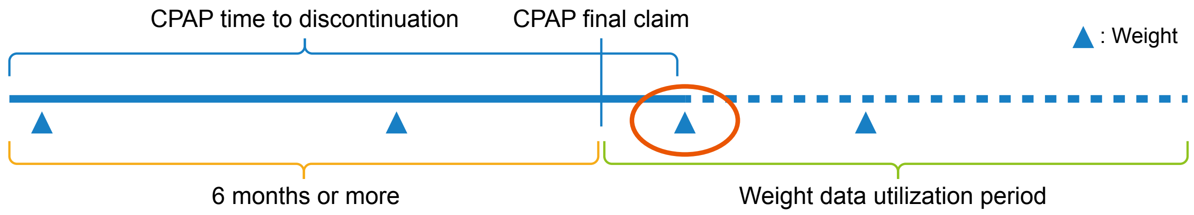

   B) Patients who did not discontinue from CPAP therapy (continued or censored)
      The data on the CPAP time to discontinuation from 180 days after the index date are used if patients persist with CPAP therapy for 180 days or more after the index date and data are available during that period. The data closest to the last claims date are used if data are available for two or more points.

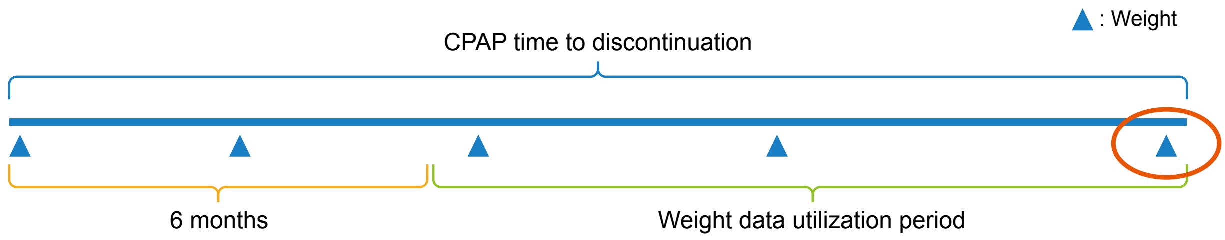

Evaluation of weight

Descriptive statistics are calculated for patients who discontinued from CPAP therapy and patients who did not discontinue CPAP therapy for the measured values at each time point and the percent change from baseline. The percentages of achievement of −10%, −20%, and −30% are calculated for weight change after the index date.

CPAP, continuous positive airway pressure; *BMI*, body mass index
